# *In vitro* osteogenic and *in ovo* angiogenic effects of a family of natural origin P_2_O_5_-free bioactive glasses†

**DOI:** 10.1039/d4ra04731a

**Published:** 2024-10-30

**Authors:** Martyna Nikody, Lilian Kessels, Lizette Morejón, Matthias Schumacher, Tim G. A. M. Wolfs, Timo Rademakers, José A. Delgado, Pamela Habibovic, Lorenzo Moroni, Elizabeth R. Balmayor

**Affiliations:** a Complex Tissue Regeneration, MERLN Institute for Technology-Inspired Regenerative Medicine, Maastricht University 6229 ER Maastricht The Netherlands; b Department of Instructive Biomaterials Engineering, MERLN Institute for Technology-Inspired Regenerative Medicine, Maastricht University Maastricht The Netherlands; c Department of Paediatrics, Research Institute for Oncology and Reproduction (GROW), Maastricht University Maastricht The Netherlands; d Center of Biomaterials, University of Havana 10400 Havana Cuba; e Cell Biology-Inspired Tissue Engineering, MERLN Institute for Technology-Inspired Regenerative Medicine, Maastricht University 6229 ER Maastricht The Netherlands; f Universitat Internacional de Catalunya 08195 Barcelona Spain; g Experimental Orthopaedics and Trauma Surgery, Department of Orthopaedic, Trauma, and Reconstructive Surgery, RWTH Aachen University Hospital 52074 Aachen Germany erosadobalma@ukaachen.de

## Abstract

Bioactive glasses (BGs) belong to a group of ceramic biomaterials having numerous applications due to their excellent biocompatibility and bioactivity. Depending on their composition, properties of BGs can be finely tuned. In this study, we investigated both angiogenic and osteogenic properties of a novel family of BGs from the SiO_2_–CaO–Na_2_O system. Three BGs were synthesised from calcite minerals and silica sands extracted from natural deposits. Silica sands used for the synthesis of each glass were obtained from different depths of the deposit, resulting in a different colour and elemental composition. The composition and structural properties of the obtained BGs were determined. Direct culture of human mesenchymal stromal cells (hMSCs) with BG particles at different concentrations was used to investigate the biocompatibility as well as the osteogenic and angiogenic properties of the BGs. In addition, BGs' effect on angiogenesis was further studied in a chick chorioallantoic membrane (CAM) model. Material characterisation confirmed the amorphous character of BGs. Investigated BGs were biocompatible and stimulated early upregulation of *RUNX2*, *ALPL*, *COL1A1*, *OCN*, and *OPN*. All BGs tested in a CAM model positively influenced the number, distribution, and branching of the blood vessels. Furthermore, our study revealed that the depth of sand deposit, at which the raw material was collected, had an impact on the osteogenic and angiogenic properties of the resulting glasses. On the one hand, silica sand collected at the deepest layer of the deposit, featuring a higher content of Fe_2_O_3_ and Al_2_O_3_, originated BGs with potent stimulative capacity of osteogenic and angiogenic gene expression. On the other hand, sand with high silica content and titanium ions resulted in a glass that better supported vessel structure. The BGs presented in this study showed the potential to promote osteogenesis and angiogenesis during bone tissue regeneration, and thus, they will be further studied as part of composite materials for the development of 3D implantable scaffolds.

## Abstract 2

A natural-origin bioactive glass' ionic composition, angiogenic, and osteogenic properties correlate with the silica sand raw material deposit depth.

## Graphical abstract

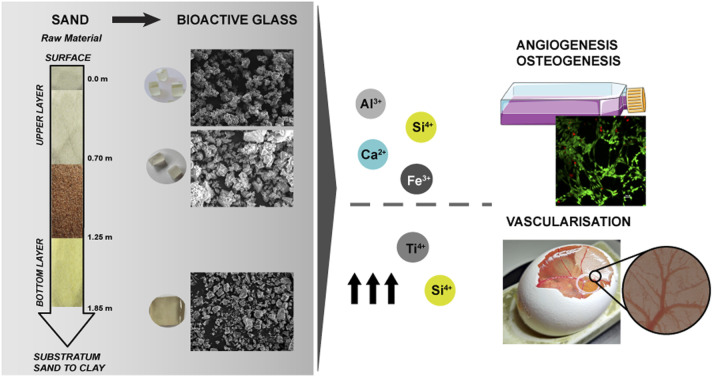

## 1 Introduction

The seminal discovery by Hench and colleagues in 1969 ^1^ that certain types of glasses were capable of forming a direct and strong bond to bone formed the basis for a still developing field. Materials capable of forming such a tight bond with natural tissues in a physiological environment have been described as bioactive.^2^ In the context of bone repair and regeneration, the bioactivity of bioactive glasses (BGs) is characterised by two main aspects: the bone bonding ability of the material itself and triggering of osteogenic differentiation facilitated by BGs' dissolution products. The underlying mechanisms of the process of bone bonding have been thoroughly investigated and reported in several publications.^3,4^ In brief, this process can be summarised in the following steps: cation exchange at the surface of the glass, silanol (Si–OH) group formation, followed by amorphous calcium phosphate phase deposition, crystallisation to hydroxycarbonated apatite (HCA), which finally binds to collagen.^2^ After the HCA layer is formed, proteins bind to the surface followed by cells. The second process through which BGs stimulate bone regeneration is mediated by the BG dissolution products that act as signals for the osteoprogenitor cells to differentiate and eventually form new bone.^5,6^ In addition, in the case of some BGs formulations, these ionic dissolution products can stimulate angiogenesis,^6,7^ which is tightly related to the development, homeostasis, and regeneration of the bone tissue.^8^

BGs belong to amorphous ceramic materials. The main classes of BGs include silicate-based, phosphate-based, and borate-based glasses.^4^ By altering the amount and proportions of the main components, that is SiO_2_, Na_2_O, and CaO, properties of BGs can be varied. Additionally, properties of BGs can be also tailored by adding or removing elements like phosphorus, borate, or strontium.^9^ Interestingly, BGs containing P_2_O_5_ as the glass-forming oxide have been reported to undergo rapid hydrolysis, making them more suitable for temporary implants used for soft tissue regeneration.^10^

The original BG (termed 45S5 and referred to as Bioglass®, which is its trademark name) was the first BG to reach the market. Several 45S5-based products have been successfully used in clinical settings mainly in the field of orthopaedics and dentistry. Its application is now being extended to soft tissue regeneration and drug delivery.^11^ Some disadvantages of the original Bioglass® have been reported, driving the research into new glass formulations. Bioglass® was reported to have relatively low dissolution and resorption rates, suboptimal thermal properties (glass transition temperature very close to the temperature of the crystallisation onset), and to cause a relatively high increase of the pH of the environment upon dissolution, contributing, for example, to cytotoxicity in cell culture experiments.^12^ Recent research efforts were expended to address these issues and develop new BG formulations for the regeneration of challenging bone defects, whereby bone-bonding properties, stimulatory osteogenic effects as well as pro-angiogenic properties are relevant.

In this study, we present novel BGs derived from silica sands and calcite extracted from natural resources. Notably, in this SiO_2_–CaO–Na_2_O system-based BGs, P_2_O_5_ is not present in the composition of the glass. Previous research,^13^ explored the potential of silica sands and calcite minerals as natural resources for the fabrication of BGs. The raw materials as well as synthesised BGs were previously characterised. Moreover, to investigate the potential application of these BGs in the field of bone regeneration, we focused on their osteogenic and angiogenic properties using *in vitro* and *in ovo* models. Possible correlations of these functional BG properties with the silica raw material used during fabrication and with the concentration of the glasses were considered.

## 2 Materials and methods

### 2.1. BGs production

**Fig. 1:**
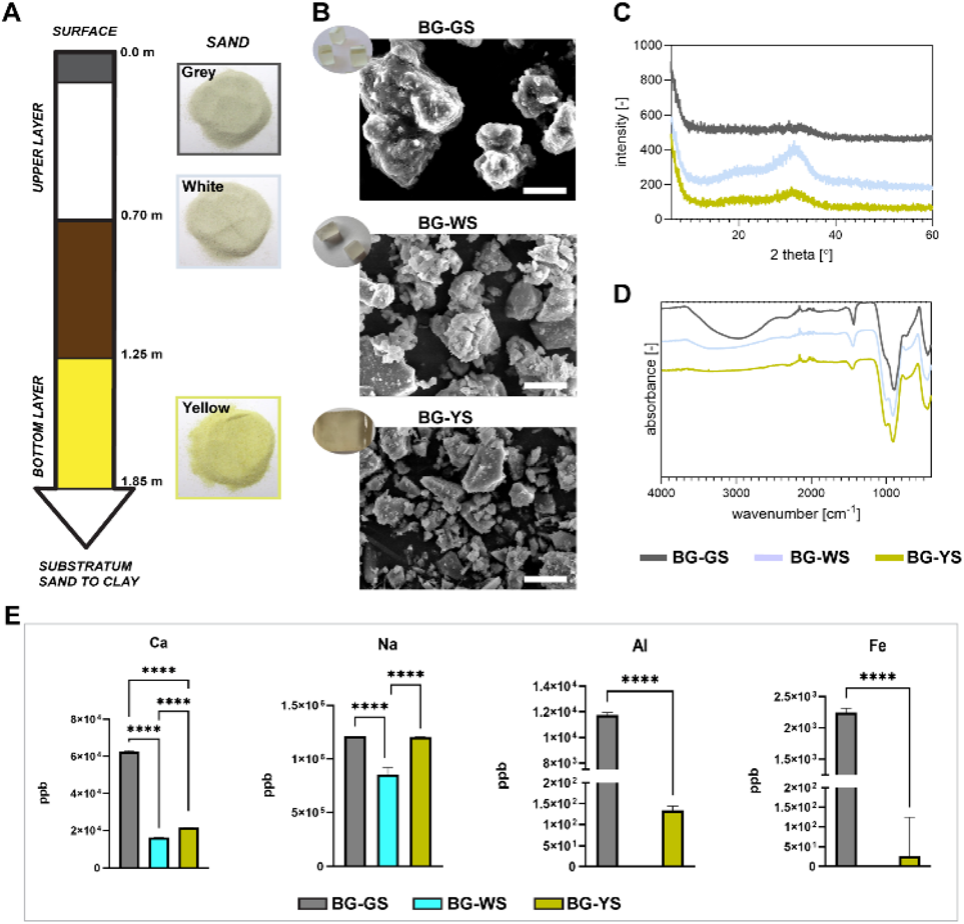
Chemical and structural characterisation of BGs investigated in the study. (A) Natural silica sand was used as a raw material; the scheme depicts the specific layer used for harvest as well as the appearance of the sand. (B) SEM images showing the morphology of the obtained BG particles. Inserted photographs depict the appearance of the glass blocks used to obtain the powders. Scale bars equal to 5 μm. (C) XRD and (D) FTIR spectra for all three BGs investigated. (E) Release of calcium (Ca), sodium (Na), aluminium (Al), and iron (Fe) ions from the BG particles investigated. Ion release was performed by incubation of the BG particles in PBS solution for 3 days at 37 °C.

Detailed production of BGs was previously described.^13^ Briefly, the BGs were prepared using silica sands extracted from the Santa Teresa deposit in Pinar del Río Province in Cuba and calcite collected from a deposit located in Jaruco in the Mayabeque Province in Cuba. Sodium carbonate anhydrous, Na_2_CO_3_ (99.5%, PanReac AppliChem, Spain) was added as a flux material and as a network modifier. All ingredients were mixed and underwent the following heating treatment: they were ramped up to 650 °C at 2 K min^−1^, ramped up to 950 °C at 1 K min^−1^, ramped up to 1450 °C at 5 K min^−1^ and held for 3 h at 1450 °C before the subsequent quench of the melt on preheated stainless-steel plates at 400 °C. Finally, the glasses were annealed for 2 h at 450 °C and cooled down slowly. Silica sands were extracted from three different depths of the deposit resulting in materials with different appearances in colour: grey sand (GS, superficial sand), white sand (WS, quartzose sand, up to 0.7 m deep), and yellow sand (YS, quartzose sand, 1.25–1.85 m deep). The corresponding glasses have been termed BG-GS, BG-WS, and BG-YS to indicate the type of silica sand material that originated each particular glass. Fig. 1A illustrates the different layers of sand from surface to clay (deepest layer), along with the layer thickness and the sand appearance. The obtained glasses were characterized by a general composition of 51.5 SiO_2_ – 23.1 CaO – 23.5 Na_2_O (wt%).^13^ The presence of the following trace elements was confirmed, Fe_2_O_3_, MnO, TiO_2_, K_2_O, P_2_O_5_, Al_2_O_3_, and MgO; all in values lower than 0.17 wt%, except for Al_2_O_3_ in BG-YS that reached 0.258 ± 0.001 wt%.^13^ Furthermore, BG-YS featured the highest content of Al_2_O_3_ and Fe_2_O_3_, while BG-GS was reported to have the highest content of TiO_2_. The glasses were obtained as solid blocks as illustrated in Fig. 1B (inserted photograph). For the present study, a powder was produced from all three glasses using a ball mill (Qiagen, Germany). Particle size was in the micrometer range; the largest observed particles featured an average of 10 μm in diameter.

### 2.2. BGs characterisation

A scanning electron microscope (SEM) (Jeol JSM-IT200 InTouchScopeto, Japan) was used to assess the morphology of the BGs in powder form. Prior to imaging, samples were mounted on aluminium stubs and sputter coated with a gold layer using a C150TES sputter coater (Quorum, United Kingdom). The chemical composition BGs was analysed by Fourier-transform infrared spectroscopy (FTIR, Nicolet iS50FT-IR, Thermo Scientific, USA) running 32 scans between 400 and 4000 cm^−1^ with a resolution of 0.5 cm^−1^. Furthermore, X-ray diffraction (XRD, D2 phaser, Bruker, USA) was performed to investigate the crystallinity using CuKα radiation (*λ* = 1.5406 Å) in the range of 2*Θ* ≤ 60° in increments of 0.01°. To investigate the release of ions from the BG particles, inductively coupled plasma mass spectrometry (ICP-MS, iCaP Q, Thermo Fisher, USA) was employed. For this, BG particles were incubated in PBS at 10% (w/v) to obtain a solution containing leachables and ions released from the material. Prior to incubation, BG particles were sterilised followed by several washing steps. The BG particles were incubated in PBS at 37 °C for three days. The resulting solutions were stored frozen until further analysis. Prior to the measurement, samples were thawed at room temperature, vortexed and centrifuged in order to remove any particles. Then, samples were diluted (1 : 80) in the previously prepared solution of nitric acid with scandium. Standards and known concentrations of a multi element solution were prepared and measured along the investigated samples.

### 2.3. Biological assessment of BGs

#### 2.3.1. *In vitro* cell culture with BG particles

Human mesenchymal stromal cells (hMSCs) used in this study were isolated from bone marrow aspirates obtained from one donor, who has given written consent. The studies involving human participants were reviewed and approved by the Medical Ethical Committee (METC) of the Maastricht University Medical Center (#15-4-274). hMSCs were cultured in basic culture medium composed of α-MEM (32561094; Thermo Fisher, the Netherlands) supplemented with 10% Fetal bovine serum (048B16; VWR, The Netherlands), and 0.2 mM ascorbic acid (1713265-25-8; Sigma-Aldrich, Germany) at 37 °C under 5% CO_2_. The culture medium was changed 24 h after seeding and twice a week during expansion.

Culture with BGs was performed in the above-mentioned growth medium with addition of 100 U per mL penicillin +100 mg per mL streptomycin (T3924; Thermo Fisher) and, in the case of positive osteogenic control, with addition of 10 nM Dexamethasone (50-02-2; Sigma-Aldrich). While assessing the influence of BGs on hMSCs, the cells were cultured in direct contact with BG particles. Prior to the start of the culture, BG particles were sterilised by treatment with 70% ethanol for 30 min repeated three times. Next, the BG particles were rinsed with sterile water and incubated overnight in culture medium at 37 °C under 5% CO_2_. Cells were seeded at density of 2.1 × 10^4^ cells per cm^2^ with selected concentrations of BG particles as indicated below. Medium change was performed twice a week. To avoid disturbing and accidently removing of the BG particles during medium change, only 80% of medium was carefully removed and then replenished with the same amount of fresh medium.

Live/dead assay, DNA-, and ALP- quantifications were performed with all three investigated BG formulations (*i.e.*, BG-GS, BG-WS, and BG-YS) at the concentrations of 100, 200, 300, 400, and 1000 μg mL^−1^. This concentration range was selected from previous investigations where we found that BGs concentrations higher than 1000 μg mL^−1^ are cytotoxic for cells in culture.^14^ Based on the findings of this study, subsequent gene expression analysis was performed at two chosen concentrations (*i.e.*, 200 and 400 μg mL^−1^).

#### 2.3.2. Live/dead assay

Cell morphology and viability were assessed through a fluorescence microscopy-based live/dead assay (L3224; Thermo Fisher) at days 1, 7, and 14 of culture. At each time point, the medium was carefully removed, samples were washed with PBS and incubated for 20 min with a solution containing 2 μM calcein AM and 4 μM EthD-1. Thereafter, the staining solution was removed, cells were washed with PBS and fresh media was added. Images were obtained with a Nikon Eclipse Ti-E microscope (Nikon Instruments Europe BV, the Netherlands) with a 10× objective. Untreated samples and samples treated with 3% DMSO were used as negative and positive controls for the assay, respectively.

#### 2.3.3. DNA quantification assay

Cell proliferation was assessed through quantification of the total DNA content at days 1, 7, and 14 by using CyQUANT® Cell Proliferation Assay Kit (C7026; Thermo Fisher) following the manufacturer's instructions. Besides experimental samples, untreated and 3% DMSO-treated cells were used as negative and positive controls for the assay, respectively. Briefly, at each time point, the culture medium was removed, and cells were thoroughly washed with PBS and stored overnight at −80 °C. To obtain cell lysates, samples were freeze-thawed three times. After that, a lysis buffer was added (0.1 M KH_2_PO_4_, 0.1 M K_2_HPO_4_, 0.1% Triton X-100, pH 7.8) and samples were incubated at room temperature for 1 h. Before proceeding, 50 μL from each sample was collected for the ALP measurement. The remaining samples were further treated with a proteinase K solution (39450-01-6; 1 mg mL^−1^; Sigma-Aldrich) in Tris/EDTA buffer and incubated overnight at 56 °C. Next, samples were freeze-thawed three times and incubated for 1 h at room temperature with a lysis buffer from the kit containing RNase (EN0531; Thermo Fisher). Finally, to measure the DNA concentration, 100 μL of the lysates was added to a black 96 well plate followed by 100 μL of GR-dye solution. Fluorescence was measured using a CLARIOstar Plus microplate reader (BGM Labtech, Germany) at emission wavelength 520 nm and excitation wavelength 480 nm. The DNA concentration was calculated based on a standard curve.

#### 2.3.4. ALP activity

Samples collected during specimen preparation for the DNA quantification were used to assess the ALP activity. 10 μL of each sample was added to a white bottom 96 well plate, followed by 40 μL of a CDP-Star® reagent (160081-62-9; Merck KGaA, Germany). After 15 min of incubation, luminescence was measured at 466 nm using a CLARIOstar Plus microplate reader (BGM Labtech). Obtained results were normalised to the DNA content.

#### 2.3.5. Gene expression

Gene expression analysis was performed to evaluate the influence of the BGs on osteogenesis and angiogenesis. The sequences of used primers are summarised in Table 1. Total RNA was isolated from hMSCs cultured with BGs at days 3, 7, and 14. RNA isolation was performed using TRIZOL reagent (15-596-018; Fisher Scientific), chloroform, and isopropanol. After isolation, the RNA pellet was resuspended in RNAse-free water (11-538-646; Fisher Scientific) and the concentration was measured using a μLITE instrument (BioDrop Ltd, USA). cDNA was synthesised using an iScript synthesis kit (Bio-Rad, USA). Expression of each gene was normalised to the housekeeping gene and the fold gene expression was calculated using the ΔΔCT method.

**Table 1:** Primer sequences of the analysed genes^a^

Gene	Forward	Reverse
*YWHAZ*	CCTGCATGAAGTCTGTAACTGAG	GACCTACGGGCTCCTACAACA
*RUNX2*	TCAACGATCTGAGATTTGTGGG	GGGGAGGATTTGTGAAGACGG
*ALPL*	ACAAGCACTCCCACTTCATC	TTCAGCTCGTACTGCATGTC
*COL1A1*	CGGTGGTTTCTTGGTCGGT	GTGCGATGACGTGATCTGTGA
*OCN*	TGAGAGCCCTCACACTCCTC	CGCCTGGGTCTCTTCACTAC
*OPN*	GTATGCACCATTCAACTCCTCG	GAAGTTTCGCAGACCTGACAT
*VEGFA*	ATCTTCAAGCCATCCTGTGTGC	GCTCACCGCCTCGGCTTGT

a Abbreviations: *YWHAZ* (tyrosine 3-monooxygenase/tryptophan 5-monooxygenase activation protein zeta), *RUNX2* (runt-related transcription factor 2), *ALPL* (alkaline phosphatase), *COL1A1* (collagen type 1 alpha 1 chain), *OCN* (osteocalcin), *OPN* (osteopontin), and *VEGFA* (vascular endothelial growth factor A).

#### 2.3.6. *In ovo* CAM assay

A CAM assay was used to investigate and compare the effect of the three BGs in terms of bioactivity and angiogenesis. For this, BG particles were incubated in PBS at 10% (w/v) to obtain a solution containing leachables and ions released from the material. The choice of this concentration was based on our previous study using a zebrafish model.^14^ Prior to incubation, BG particles were sterilised followed by several washing steps. The BG solutions were incubated at 37 °C for three days. Prior to the start of the CAM assay, the solutions were centrifuged and the collected supernatant was sterilised by passing it through a 0.2 μm filter. PBS only as well as PBS with the addition of 10 ng of VEGFA (130-109-384; Miltenyi Biotec, Germany) served as negative and positive control, respectively. Fertilised chicken eggs were provided by Het Anker B.V. (the Netherlands). Eggs (*n* = 50) were kept in an incubator at 37 °C under 50–55% humidity and rotated once an hour. After three days, windows of the dimensions of 1 × 1.5 cm^2^ were carefully cut into the eggshells and covered with adhesive tape. On day 10, silicone rings were carefully placed on the chorioallantoic membranes and the previously prepared solutions were added to the area inside the rings. Solutions containing PBS (control), PBS + VEGF (control), PBS + BG-WS, PBS + BG-YS, and PBS + BG-GS were added to the membranes of selected embryos. *n* = 10 embryos were assigned to each of the investigated groups, including controls. In the BG-GS group, *n* = 3 embryos were lost for causes not related to the study before the sample application. Embryos were returned to the incubator for further four days. Thereafter, on day 14 of incubation, CAM membranes were imaged using a digital measuring microscope (DMS 300, Leica, Germany) as well as an automated stereomicroscope (SMZ25, Nikon Instruments Europe BV). Images were acquired at a zoom of 1×, 3×, and 5×, and the 3× images were used for quantification. Between 6 and 10 representative pictures per studied group were used for quantification purposes. Images were analysed using NIS Elements AR GA3 module (Nikon Instruments Europe BV) using a custom-built pipeline. The following parameters were then extracted: surface coverage (vessel area/total image area; ‘Object Area’, %), total vascular length (‘Length’, μm), number of branches (‘Object Count’), and number of end points (‘Object Count’). From these parameters, the branching/vessel length (μm^−1^) and the end points/vessel length (μm^−1^) were calculated.

### 2.4. Statistical analysis

Statistical analysis of the results was performed with GraphPad prism software (GraphPad 10.1.0). Analysis of the DNA and ALP assay was performed using 2way ANOVA followed by Tukey's multiple comparison test. Two replicates per condition were analysed. Analysis of qPCR data was performed using ordinary one-way ANOVA followed by Tukey's multiple comparison test. Three replicates were analysed. Quantitative results of the CAM assay were analysed with Ordinary one-way ANOVA followed by Bonferroni's multiple comparison test. Between 6 and 10 replicates were used for the analysis. Values were considered significant at *p* < 0.05.

## 3 Results

### 3.1. Characterisation of bioactive glasses

Three different BGs were obtained using natural sand and calcite minerals as starting, raw materials. The glasses shared the same general composition (*i.e.*, 51.5 SiO_2_ – 23.1 CaO – 23.5 Na_2_O (wt%)) but differed in trace element content and appearance (Fig. 1B). For the study, a fine powder was produced and characterized by SEM. The morphology of the BG particles can be observed in Fig. 1B. Particles of the three BG formulations appeared irregular in shape. In order to characterise the crystallinity and chemical composition of the materials, XRD and FTIR were performed (Fig. 1C and D). FTIR spectra revealed the main reflexes around 980 and 1040 cm^−1^, characteristic of the Si–O bonds (Fig. 1D). Furthermore, the broad band around 3000 cm^−1^, typically corresponding to absorbed water, revealed a hygroscopic character of, in particular, BG-GS. The absence of sharp peaks on XRD patterns confirmed the amorphous character of the BGs (Fig. 1C). Yet, minor peaks on the broad band observed on the diffractogram at 2theta 30°–40°, may suggest the formation of crystalline phases (*e.g.*, combeite, Na_2_Ca_2_Si_3_O9). This was observed in all formulations but was most pronounced in the case of BG-WS. In addition, ICP-MS measurement revealed different release profiles of Ca, Na, Al, and Fe after 3 days of incubation of BG particles in PBS (Fig. 1E). Overall, BG-GS particles released the highest amount of the measured ions among the three investigated BGs. Release of Al and Fe was below detection levels in PBS solutions incubated with BG-WS particles.

### 3.2. Viability and morphology of hMSCs in contact with different BGs

**Fig. 2:**
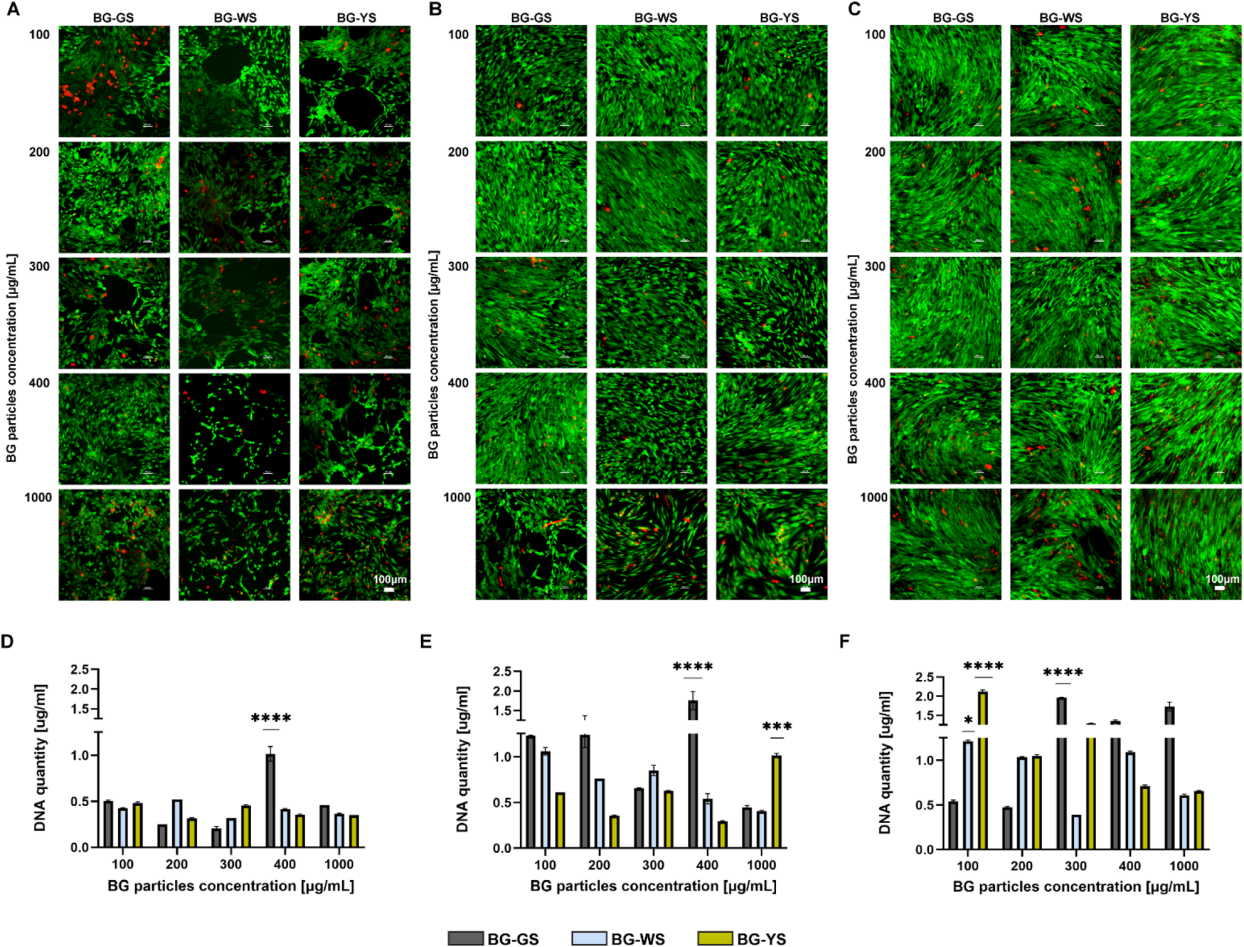
Live/dead staining showing viability as well as morphology of hMSCs cultured with different BGs at varied concentrations for (A) 1 day, (B) 7 days, and (C) 14 days. Scale bars are equal to 100 μm. Calcein AM stained the viable cells green while EthD-1 labelled the non-viable cells red. DNA content on day 1 (D), 7 (E), and 14 (F) of cell culture.

To assess the effects of BGs on hMSCs, the cells were cultured in direct contact with the BG particles. Live/dead cell imaging showed that the highest tested BG concentration, 1000 μg mL^−1^, had certain toxicity on the cells, as indicated by either a large number of dead cells or by empty spaces caused by the washing out of the dead cells (Fig. 2A–C). Lower concentrations (100–400 μg mL^−1^) supported cell viability and proliferation for up to 14 days of culture. Interestingly, on day 1 (Fig. 2A), the cell morphology was altered in the presence of the BG particles, in particular BG-WS and BG-YS. Here, the cells seemed to arrange themselves in tubule-like structures. Analysis at later time points, day 7 (Figure 2B) and 14 (Fig. 2C), did not reveal any obvious differences in cell morphology between the tested conditions as well as the untreated control (Fig. S2†) due to high cell confluence. Quantification of DNA (Fig. 2D–F and S1†) over the period of 14 days of direct culture of hMSCs with the three BGs showed an overall increase in the DNA content in the presence of all three BGs. The highest DNA content was detected on day 14 when cells were cultured with BG-GS at 300 μg mL^−1^ or BG-YS at 100 μg mL^−1^. On day 1 and 7 of culture, BG-GS at concentration of 400 μg mL^−1^ induced the highest cell proliferation. In the case of culture with BG-WS on day 7, concentration of 100 μg mL^−1^ resulted in the highest DNA content, however, no statistically significant differences were observed compared to the other two BGs. The highest concentration used of BG-YS resulted in the highest proliferation on day 7. Finally, on the last time point post-culture (*i.e.*, day 14), the lowest tested concentration (*i.e.*, 100 μg mL^−1^) resulted in the highest DNA content in the case of BG-WS and BG-YS. In the case of BG-GS, a concentration of 300 μg mL^−1^ resulted in the highest cell proliferation.

### 3.3. Influence of different concentrations of BGs on ALP activity of hMSCs

**Fig. 3:**
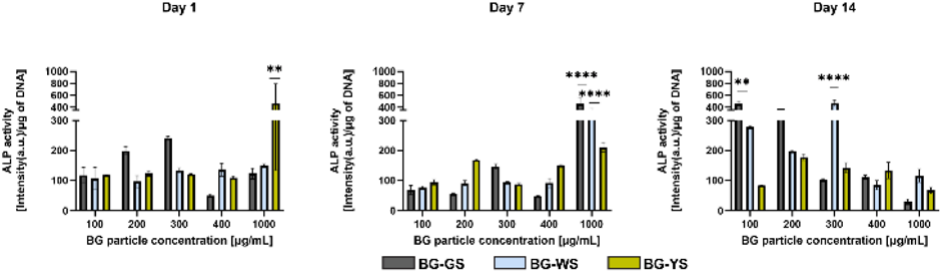
Effects of BG-GS, BG-WS, and BG-YS and at a range of concentrations (100–1000 μg mL^−1^) on ALP activity of hMSCs on days 1, 7, and 14 of cell culture.

ALP activity (Fig. 3) was measured on day 1, 7, and 14 of culture. On day 1, the highest ALP activity was observed for 1000 μg mL^−1^ of BG-YS. Interestingly, BG-GS stimulated ALP activity on hMSCs only at low concentrations with the highest activity noted in the presence of 300 μg mL^−1^. BG-WS did not show a revelatory effect on the ALP activity of hMSCs for this time of observation; no major differences in ALP activity were detected when comparing the different concentrations of BG-WS used. On day 7, the highest ALP activity was observed at the highest tested concentration for all three BGs, with BG-GS and BG-WS showing a most pronounced effect at 1000 μg mL^−1^. On day 14, BG-GS stimulated the highest ALP activity in hMSCs when used at 100 μg mL^−1^ while BG-WS was most stimulative at 300 μg mL^−1^. BG-YS showed a comparable effect on ALP for all tested concentrations, that was always inferior when compared to the other two glasses.

Based on these results and those obtained from the biocompatibility assessment, concentrations of 200 and 400 μg mL^−1^ were chosen for further evaluations of the osteogenic and angiogenic properties of the three types of BG.

### 3.4. Influence of BGs on osteogenesis and angiogenesis at the mRNA level

**Fig. 4:**
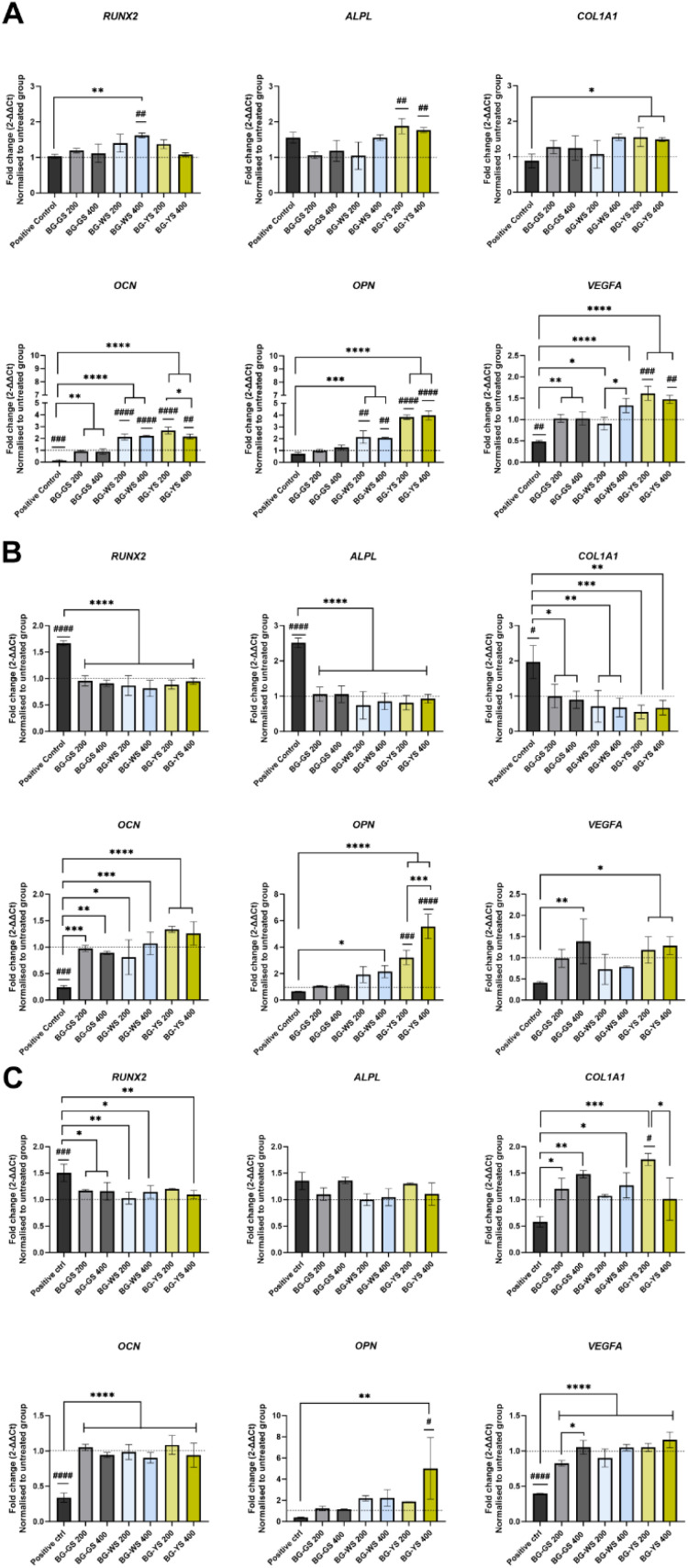
Expression of the osteogenic gene markers *RUNX2*, *ALPL*, *COL1A1*, *OCN*, and *OPN* as well as an angiogenic gene marker *VEGFA* upon culture of hMSCs with different BGs at 200 or 400 μg mL^−1^. (A) day 3, (B) day 7, and (C) day 14. Fold gene expression was calculated using ΔΔCT method. Results are normalised to the control group indicated by the dashed line (*i.e.*, cells cultured in basic medium without BG particles). [*] marks significant differences compared to the positive control group as well as differences between the same BG at two concentrations. [#] symbols indicate a statistically significant difference between the control group and the rest of the conditions.

Expression of *RUNX2*, *ALPL*, *COL1A1*, *OCN*, *OPN*, and *VEGFA* was assessed after 3 (Fig. 4A), 7 (Fig. 4B), and 14 days (Fig. 4C) of direct culture with three BGs at either 200 or 400 μg mL^−1^. Comparison between the untreated group (*i.e.*, cells cultured in the absence of BGs in basic medium), positive control (*i.e.*, cells cultured in the absence of BGs in basic medium supplemented with Dexamethasone), and direct cell culture with three different BG particles in basic medium at two concentrations revealed several differences.

Expression of *RUNX2* on day 3 was significantly upregulated in the case of culture with BG-WS at 400 μg mL^−1^, compared to both the untreated group and the positive control. At later time points, *i.e.*, day 7 and 14 of culture, the expression of *RUNX2* was the highest for the positive control, while the expression in the presence of BGs was comparable to the untreated control.

*ALPL* was upregulated in the case of the culture with BG-YS at both concentrations on day 3. Upregulation was statistically significant when compared to the untreated control but not to the positive control. On day 7, *ALPL* expression of cells cultured in the presence of either BG-GS, BG-WS, or BG-YS was significantly lower than in case of the positive control and comparable with the untreated control. On day 14, no statistically significant differences were observed among any of the conditions.

Expression of *COL1A1* on day 3 in the presence of BG-WS at 400 μg mL^−1^ and BG-YS at 200 μg mL^−1^ was statistically higher than the positive control. On day 7, a similar trend to the expression of *RUNX2* and *ALPL* was observed, *i.e.*, expression in the presence of BGs was lower than in the positive control, but comparable with the untreated control. On day 14, expression of *COL1A1* was higher in the presence of all three BGs tested when compared to the positive control. Statistically relevant differences were found for 200 μg mL^−1^ and for 400 μg mL^−1^ of BG-GS, 400 μg mL^−1^ of BG-WS, and for 200 μg mL^−1^ of BG-YS. The latter condition stimulated the highest COL1A1 expression on hMSCs among all samples and controls evaluated.

*OCN* on day 3 was upregulated in case of BG-WS and BG-YS at both tested concentration with statistically significant differences when compared with both the untreated group and the positive control. Furthermore, cells cultured with BG-GS at both concentrations showed a significantly higher *OCN* expression than the positive control. Expression was higher in case of culture with all BG conditions, however, no statistically significant differences were observed in comparison with the untreated group. Later times of observation showed a significantly higher *OCN* expression for all the glasses, unrelated to the concertation used, when compared to the positive control.

*OPN* was upregulated on day 3 in cells cultured with either 200 μg mL^−1^ or 400 μg mL^−1^ of BG-WS compared to both the untreated group and the positive control. Similar results were obtained for both concentrations of BG-YS. *OPN* expression remained unchanged up to day 7 of culture. The higher concentration of BG-YS stimulated upregulation of *OPN*, while only BG-WS at 400 μg mL^−1^ remained upregulated when compared to the positive control. On day 14, only BG-YS at 400 μg mL^−1^ resulted in upregulation of *OPN* when compared to both, the untreated and the positive control.

Expression of the angiogenic marker, *VEGFA*, was higher for BG groups when compared to the positive control at all time points. On day 3 of culture, the highest upregulation of *VEGFA* resulted from the culture with BG-YS, which was also significantly higher when compared with the untreated group. On day 7, no condition resulted in higher *VEGFA* expression when compared with the untreated control, but BG-GS at 400 μg mL^−1^ and BG-YS at both concentrations stimulated *VEGFA* upregulation when compared to the positive control. This effect was sustained until day 14 with the mentioned BGs.

### 3.5. Influence of BGs on the vasculature of the CAM

**Fig. 5:**
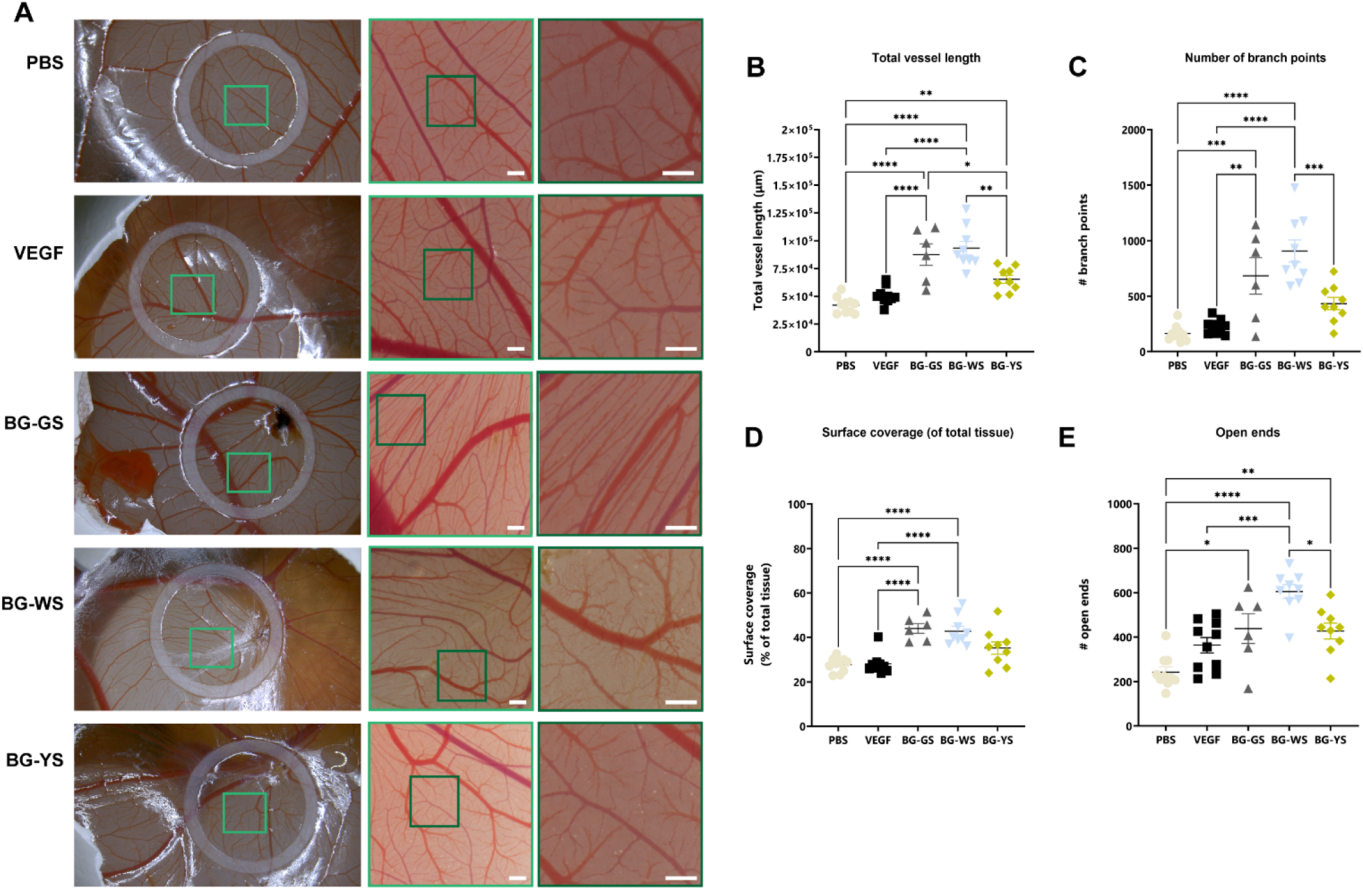
*In ovo* CAM assay results. (A) Representative macro- and microscopy images of the CAM assay for the control (*i.e.*, PBS as negative control and VEGFA as positive control) and for the BG experimental groups after an incubation time of 4 days post-addition of the solutions to the CAM. Scale bars equal 500 μm. (B–E) quantification of the obtained results comparing (B) total vessel length, (C) number of branch points, (D) surface coverage, and (E) open ends.

A CAM assay was used to test the influence of BGs on angiogenesis. For this purpose, PBS solutions conditioned by BGs were prepared. Differences in the amount of vasculature were visible (Fig. 5A) at the macroscopic level and were confirmed through quantification the total vessel length (Fig. 5B), number of branch points (Fig. 5C), surface coverage (Fig. 5D), and number of open ends (Fig. 5E). Total vessel length as well as the number of open ends were significantly higher for all three tested BGs in comparison with the PBS control. Total vessel length, surface coverage of the total tissue, and number of branching points were significantly higher when exposed to BG-GS and BG-WS in comparison to both, PBS and VEGFA controls. In addition, the number of branching points as well as the number of open ends quantified on CAM vasculature exposed to BG-WS were significantly higher than in the case of BG-YS. Furthermore, all the quantified parameters were the highest and the most consistent when BG-WS was used. Interestingly, when comparing macroscopic appearance of the CAM vasculature among all the BGs, a certain degree of directionality was observed in the BG-GS condition.

## 4 Discussion

Successful treatment of critical-sized bone defects remains a clinical challenge, motivating research into effective biomaterials-based alternatives for autologous bone grafts. In this context, BGs are an interesting class of biomaterials; they often feature not only desired osteogenic properties but also angiogenic and antibacterial characteristics. In this study, we investigated three BGs formulations from the SiO_2_–CaO–Na_2_O system that were synthesised from natural silica sands and calcite mineral. The synthesis and characterisation of these glasses was previously reported by our group.^13^ Here, the osteogenic and angiogenic effects of the glasses were investigated using *in vitro* and *in ovo* models.

To test the effect of the BGs on human cells, we employed a direct *in vitro* culture of clinically relevant hMSCs with BG particles at a broad range of concentrations. All tested formulations of BGs showed good biocompatibility, although the highest tested concentration of 1000 μg mL^−1^ caused a certain toxicity effect on the cells. Interestingly, cells formed arrangements of tubule-like structures in the presence of BG-WS and BG-YS. Decker *et al.*^15^ showed similar findings when applying dissolution products of boron-doped BGs from the SiO_2_–CaO–Na_2_O–P_2_O_5_–K_2_O system on bone marrow-derived MSCs. In that study, the change in cell morphology was observed from day 3 onwards, which is later than our observation on day 1. This difference may result from the different experimental set up used, *i.e.*, direct culture in the presence of BGs *versus* indirect culture by exposing the cells to BG dissolution products. The fact that we observed an accelerated effect on cell morphology by the BG may be related to the direct material–cell interaction, responsible not only for the influence of dissolution products but also for the material's topography and structure. Bellucci *et al.*^16^ stressed that it is essential to integrate the results of direct culture methods with those from the indirect approach in the biocompatibility assessment of BGs. The observed change in cell morphology, *i.e.*, the formation of tubule-like structures, might suggest an angiogenic response of hMSCs to the BGs.

In addition to differentiating into osteoblasts, chondrocytes and adipocytes, hMSCs have also been reported to be able to differentiate into cells of the endothelial lineage.^17^ Analysis of the expression of *VEGFA* at the mRNA level indeed confirmed the effect of the BGs, as BG-YS provoked early upregulation of *VEGFA* expression. Existing literature reports similar findings to our study. For instance, cobalt-doped borosilicate glasses were shown to upregulate the expression of several angiogenic gene markers, including *VEGFA*, in hMSCs in a concentration-dependent manner.^18^ In a similar study, increased *VEGFA* expression by hMSCs was reported after culture with copper-doped 45S5 based BGs.^19^ Another example is the study by Azevedo *et al.*,^20^ where cobalt-releasing BGs from the SiO_2_–P_2_O_5_–CaO–Na_2_O–CoO system were shown to upregulate *VEGFA* expression in a concentration-dependent manner. As is evident from these examples, the majority of the BGs that have shown an effect on angiogenesis, contained specific elements, such as cobalt or copper, known for being angiogenesis stimulators. In the present study, the BG synthesised from yellow sand, BG-YS, showed the strongest effect on *VEGFA* expression. The reason for this might be a higher content of Fe_2_O_3_ in this particular sand,^13^ as literature reports indicate a pro-angiogenic character of Fe_2_O_3_-containing biomaterials.^21,22^

Earlier studies have shown that *in vitro* evaluation of the angiogenic properties of BGs may provide variable readouts, highly dependent on the cell type used.^23,24^ Hence, the information obtained is often limited. A more complex evaluation platform to study angiogenesis is the CAM assay. The CAM is a highly vascularised membrane surrounding a developing chicken embryo. During embryonic development, it is responsible for gas exchange, waste product management, and calcium transport from the eggshell.^25^ In addition, the CAM can be considered an immunodeficient environment, as the chicken acquires a mature immune system just before hatching.^26^ In comparison with *in vitro* assays, the CAM assay offers a more complex environment to study angiogenesis. Therefore, the effect of the three BG types on angiogenesis was further investigated in an *in ovo* CAM assay. Overall, all tested BGs influenced the vasculature of the CAM, however, BG-GS and BG-WS had a stronger overall effect than BG-YS. This was surprising, as BG-YS, synthesised with yellow sand was the most potent inductor of *VEGFA* expression on hMSCs in *in vitro* culture. The observed differences in angiogenic effect seem to be related to the silica sand composition, which was found to be dependent on the depth of the sand deposit. In particular, the presence of TiO_2_ and high silica content seemed to govern the angiogenic potential of the glass. This is not surprising; the stimulatory angiogenic properties of silica-based materials have been documented by Dashnyam *et al.*,^7^ and Kargozar *et al.*,^10^ and of titanium have been demonstrated in studies from Martins *et al.*^27^ and Khalid *et al.*^28^

Similar behaviour was observed regarding the osteogenic character of these materials. All three BGs positively impacted the osteogenic differentiation of hMSCs, although gene expression was found to be dependent on the composition of the glass. Tested BGs appeared to influence osteogenic differentiation despite the absence of P_2_O_5_ in their network. P_2_O_5_ in the glass network is a source of phosphorus, which is required for hydroxyapatite formation during the process of mineralization in the skeletal system. However, the presence of P_2_O_5_ has been reported to contribute to increased hydrolysis, making the glasses of this system more suitable for soft tissue regeneration as temporary implants.^10^ Li *et al.*,^24^ reported that higher phosphorus content in BGs might promote more rapid glass degradation. Nevertheless, several BGs without phosphorus in their network have been reported to support osteogenic differentiation.^14,29^ Further advantages of the P_2_O_5_-free glasses include antibacterial properties.^30–32^ Based on the above-mentioned advantages, P_2_O_5_-free bioactive glasses may be more adequate for hard tissue regeneration than their P_2_O_5_-enriched counterpart. Here, we have shown that the expression of several osteogenic gene markers was upregulated more rapidly in the presence of BG particles than in the presence of Dexamethasone, a glucocorticoid with a known positive effect on osteogenic differentiation. Especially BG-WS and BG-YS resulted in early upregulation of *RUNX2*, *COL1A1*, *OCN*, and *OPN*. Similarly to the angiogenesis previously discussed, the BGs' osteogenesis seemed to be dependent on the type of sand used as a precursor for the synthesis of the glasses. Sand from the deepest layer of the deposit (*i.e.*, yellow sand) originated a BG with the strongest upregulation of several of the investigated genes while surface sand resulted in a glass with no influence on osteogenic gene expression. Here also, the observed differences might be related to the presence of different trace elements. The glass with the strongest osteogenic effect features a higher content of Fe_2_O_3_ and Al_2_O_3_. Several reports in recent literature are supportive of our correlation of the observed osteogenic properties to the presence of Fe_2_O_3_. An *in vivo* investigation using a critical-sized mandibular defect model in dogs reported increased bone formation in the presence of calcium silicate-doped Fe_2_O_3_ material.^33^ The osteogenic potential was shown to be dependent on the Fe_2_O_3_ concentration. A concentration-dependent effect was also concluded by Bai *et al.*^34^ using MSCs *in vitro*. The authors showed that osteogenic or chondrogenic differentiation could be favoured depending on the Fe_2_O_3_ concentration used. Likewise, using an *in vitro* study design, Fopase *et al.* substituted SiO_2_ with Fe_2_O_3_ in Bioglass®, which led to increased viability and proliferation of MG63 osteoblasts.^35^ Regrettably, no concrete osteogenesis evaluation was performed on the stimulated MG63, however, considering our data and that of other studies it could be expected that the Fe_2_O_3_ – Bioglass® also potentiated the osteoblastic genotype of MG63 osteoblasts. An Al_2_O_3_ dopped Bioglass® has also been reported featuring improved mechanical properties, also relevant in an osteogenic material.^36,37^ Here also, a concentration-dependent effect was described but in this case regarding the bioactivity of the glass. A high content of Al_2_O_3_ in Bioglass® was shown to be detrimental for bioactivity. Regrettably, a lack of literature exists on the effect of Al_2_O_3_ dopped glasses on cell cultures and *in vivo* testing.

Overall, it can be summarized that the three natural origin P_2_O_5_-free bioactive glasses investigated here displayed a positive effect on both osteogenesis and angiogenesis as demonstrated using an *in vitro* and *in ovo* experimental setup. The results revealed a relationship between the BGs properties and the composition of the sand used as a precursor for their fabrication. Sand collected at the deepest deposit layers, rich in Fe_2_O_3_ and Al_2_O_3_, originated a glass highly potent regarding the osteogenic and angiogenic stimulation at the gene level. Importantly, for desired vascularization—evaluated here by vessel length and number of branch points among other properties of the CAM—, the glass should feature high silica content and titanium ions. This composition was found in the white sand, collected in the upper layer up to 0.7 m in depth of the sand deposit. It might be then interesting to use a composite sand as a glass precursor obtained by mixing sands from the upper (white coloured) and bottom layer (yellow coloured) of the deposit. Future investigations using this material should also consider testing the possible antibacterial properties of the glasses. Certain BGs are known to be bactericidal, a property of relevance in the context of segmental bone defects. In addition, despite the proven biocompatibility, osteogenic, and angiogenic properties of these materials, demonstrated here *in vitro* and *in ovo*, these materials should be further validated in a more complex *in vivo* animal model.

## 5 Conclusions

Our study demonstrated that BGs from the SiO_2_–CaO–Na_2_O system, free of P_2_O_5_ and produced using natural origin raw materials as precursors may be promising biomaterials to simultaneously stimulate angiogenesis and osteogenesis in the context of challenging bone defects. Silica sands obtained from three different depths of a naturally occurring sand deposit were used to prepare the BGs. The depth of sand deposits, which determined the elemental composition of the sand, has a profound effect on the osteogenic and angiogenic properties of the resulting glasses. Sand with high silica content that contains Fe_2_O_3_, Al_2_O_3_, and TiO_2_ originates a glass that not only has an impact on the angiogenic and osteogenic gene expression but that is also capable of stimulating vasculature development.

## Data availability

Supporting, raw data for this article are available at the Dataverse repository (https://dataverse.nl/).

## Author contributions

Martyna Nikody: conceptualization, investigation, methodology, visualization, writing – original draft, writing – review & editing. Lilian Kessels: methodology. Lizette Morejón: resources. Matthias Schumacher: methodology. Tim G. A. M. Wolfs: resources. Timo Rademakers: formal analysis. José A. Delgado: resources. Pamela Habibovic: supervision, writing – review & editing, funding acquisition. Lorenzo Moroni: supervision, writing – review & editing, funding acquisition. Elizabeth R. Balmayor: conceptualization, resources, supervision, writing – review & editing.

## Conflicts of interest

There are no conflicts to declare.

## Supplementary Material

RA-014-D4RA04731A-s001
