# Supplementary material for: In vitro osteogenic and in ovo angiogenic effects of a family of natural origin P2O5-free bioactive glasses
Source: RSC Adv. 2024 Oct 30;14(47):34708–17. doi: 10.1039/d4ra04731a (PMC11523078; doi:10.1039/d4ra04731a)
Supplement: RA-014-D4RA04731A-s001 [file RA-014-D4RA04731A-s001.pdf]

Supplementary Information (SI) for RSC Advances.
This journal is © The Royal Society of Chemistry 2024

Supplementary Information

In vitro osteogenic and *in ovo* angiogenic effects of a family of natural origin P_2O_5-free bioactive glasses

Martyna Nikody,[a,b] Lilian Kessels[c], Lizette Morejón[d], Matthias Schumacher[b], Tim G.A.M. Wolfs[c], Timo Rademakers[a,b,e], José A. Delgado[d,f], Pamela Habibovic[b], Lorenzo Moroni[a], Elizabeth R. Balmayor*[e,g]

* *Corresponding author*

[a.] *Complex Tissue Regeneration, MERLN Institute for Technology-Inspired Regenerative Medicine, Maastricht University, 6229 ER Maastricht, the Netherlands.*
[b.] *Department of Instructive Biomaterials Engineering, MERLN Institute for Technology-Inspired Regenerative Medicine, Maastricht University, Maastricht, the Netherlands.*
[c.] *Department of Paediatrics, Research Institute for Oncology and Reproduction (GROW), Maastricht University, Maastricht, the Netherlands*
[d.] *Center of Biomaterials, University of Havana, 10400 Havana, Cuba*
[e.] *Cell Biology-Inspired Tissue Engineering, MERLN Institute for Technology-Inspired Regenerative Medicine, Maastricht University, 6229 ER Maastricht, the Netherlands*
[f.] *Universitat Internacional de Catalunya, 08195 Barcelona, Spain*
[g.] *Experimental Orthopaedics and Trauma Surgery, Department of Orthopaedic, Trauma, and Reconstructive Surgery, RWTH Aachen University Hospital, 52074 Aachen, Germany*

Day 1

Tukey's multiple comparisons test **BG-GS**	Adjusted P Value	Summary
100 vs. 200	0.0047	**
100 vs. 300	0.0022	**
100 vs. 400	0.0002	***
100 vs. 1000	0.7320	**ns**
200 vs. 300	0.7320	**ns**
200 vs. 400	<0.0001	****
200 vs. 1000	0.0112	*
300 vs. 400	<0.0001	****
300 vs. 1000	0.0047	**
400 vs. 1000	0.0001	***

Tukey's multiple comparisons test **BG-WS**	Adjusted P Value	Summary
100 vs. 200	<0.0001	****
100 vs. 300	<0.0001	****
100 vs. 400	0.4502	**ns**
100 vs. 1000	0.0006	***
200 vs. 300	<0.0001	****
200 vs. 400	<0.0001	****
200 vs. 1000	<0.0001	****
300 vs. 400	<0.0001	****
300 vs. 1000	0.0023	**
400 vs. 1000	0.0014	**

Tukey's multiple comparisons test **BG-YS**	Adjusted P Value	Summary
100 vs. 200	<0.0001	****
100 vs. 300	0.1371	**ns**
100 vs. 400	0.0001	***

100 vs. 1000	0.0001	***
200 vs. 300	<0.0001	****
200 vs. 400	0.0252	*
200 vs. 1000	0.0427	*
300 vs. 400	0.0004	***
300 vs. 1000	0.0003	***
400 vs. 1000	0.9695	**ns**

Day 7

Tukey's multiple comparisons test **BG-GS**	Adjusted P Value	Summary
100 vs. 200	>0.9999	**ns**
100 vs. 300	0.0266	*
100 vs. 400	0.0356	*
100 vs. 1000	0.0070	**
200 vs. 300	0.0248	*
200 vs. 400	0.0383	*
200 vs. 1000	0.0066	**
300 vs. 400	0.0015	**
300 vs. 1000	0.4891	**ns**
400 vs. 1000	0.0006	***

Tukey's multiple comparisons test **BG-WS**	Adjusted P Value	Summary
100 vs. 200	0.0038	**
100 vs. 300	0.0183	*
100 vs. 400	0.0003	***
100 vs. 1000	<0.0001	****
200 vs. 300	0.3056	**ns**
200 vs. 400	0.0150	*
200 vs. 1000	0.0018	**
300 vs. 400	0.0033	**
300 vs. 1000	0.0006	***
400 vs. 1000	0.0975	**ns**

Tukey's multiple comparisons test **BG-YS**	Adjusted P Value	Summary
100 vs. 200	<0.0001	****
100 vs. 300	0.6685	**ns**
100 vs. 400	<0.0001	****
100 vs. 1000	<0.0001	****
200 vs. 300	<0.0001	****
200 vs. 400	0.0143	*
200 vs. 1000	<0.0001	****
300 vs. 400	<0.0001	****
300 vs. 1000	<0.0001	****
400 vs. 1000	<0.0001	****

Day 14

Tukey's multiple comparisons test	Adjusted P Value	Summary

BG-GS		
100 vs. 200	0.7293	**ns**
100 vs. 300	<0.0001	****
100 vs. 400	0.0001	***
100 vs. 1000	<0.0001	****
200 vs. 300	<0.0001	****
200 vs. 400	<0.0001	****
200 vs. 1000	<0.0001	****
300 vs. 400	0.0004	***
300 vs. 1000	0.0317	*
400 vs. 1000	0.0043	**

Tukey's multiple comparisons test **BG-WS**	Adjusted P Value	Summary
100 vs. 200	0.0001	***
100 vs. 300	<0.0001	****
100 vs. 400	0.0007	***
100 vs. 1000	<0.0001	****
200 vs. 300	<0.0001	****
200 vs. 400	0.0243	*
200 vs. 1000	<0.0001	****
300 vs. 400	<0.0001	****
300 vs. 1000	<0.0001	****
400 vs. 1000	<0.0001	****

Tukey's multiple comparisons test **BG-YS**	Adjusted P Value	Summary
100 vs. 200	<0.0001	****
100 vs. 300	<0.0001	****
100 vs. 400	<0.0001	****
100 vs. 1000	<0.0001	****
200 vs. 300	0.0006	***
200 vs. 400	<0.0001	****
200 vs. 1000	<0.0001	****
300 vs. 400	<0.0001	****
300 vs. 1000	<0.0001	****
400 vs. 1000	0.2129	**ns**

Figure S1. Detailed statistical comparison of the influence of different concentrations of BG-GS, BG-WS, and BG-YS on days 1, 7, and 14 on the DNA content of hMSCs.

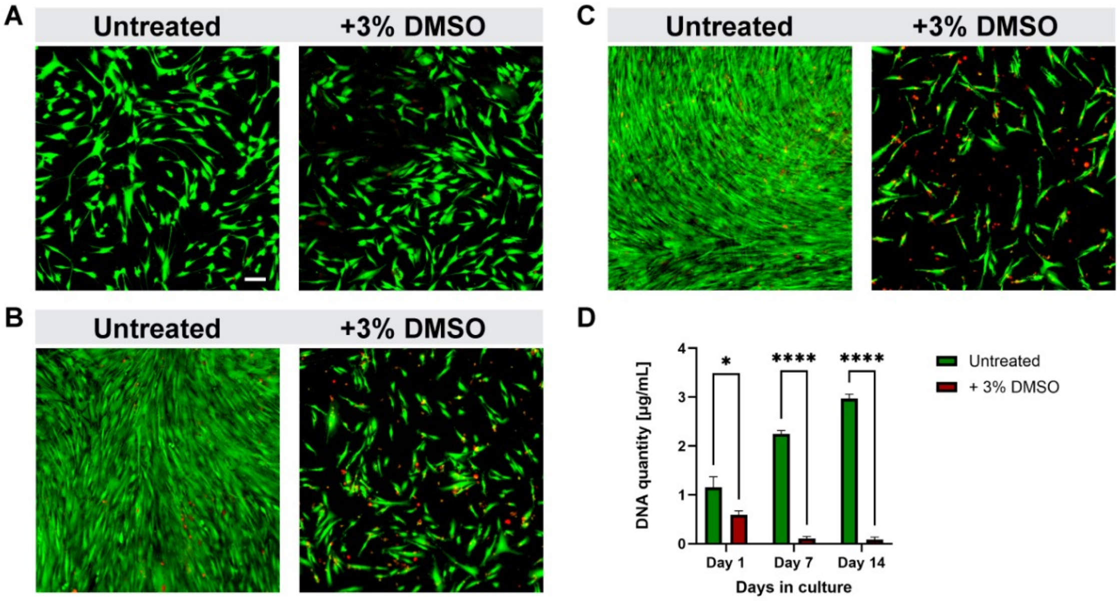

Figure S2. Live/Dead staining showing viability as well as morphology of hMSCs cultured with basic culture medium (untreated) and with basic culture medium containing 3% DMSO for A) 1 day, B) 7 days, and C) 14 days. Scale bar is equal to 100 µm. Calcein AM stained the viable cells green while EthD-1 labelled the non-viable cells red. D) DNA content on day 1, 7, and 14 of cell culture in the above-mentioned conditions.
